# Nanomedicine strategies to counteract cancer stemness and chemoresistance

**DOI:** 10.37349/etat.2023.00157

**Published:** 2023-08-30

**Authors:** Huayu Liu, Mingqi Liu, Yanan Zhao, Ran Mo

**Affiliations:** Huazhong University of Science and Technology, China; State Key Laboratory of Natural Medicines, Jiangsu Key Laboratory of Drug Discovery for Metabolic Diseases, Center of Advanced Pharmaceuticals and Biomaterials, School of Life Science and Technology, China Pharmaceutical University, Nanjing 210009, Jiangsu, China

**Keywords:** Drug delivery, nanomedicine, chemoresistance, cancer stem-like cells, cancer therapy

## Abstract

Cancer stem-like cells (CSCs) identified by self-renewal ability and tumor-initiating potential are responsible for tumor recurrence and metastasis in many cancers. Conventional chemotherapy fails to eradicate CSCs that hold a state of dormancy and possess multi-drug resistance. Spurred by the progress of nanotechnology for drug delivery and biomedical applications, nanomedicine has been increasingly developed to tackle stemness-associated chemotherapeutic resistance for cancer therapy. This review focuses on advances in nanomedicine-mediated therapeutic strategies to overcome chemoresistance by specifically targeting CSCs, the combination of chemotherapeutics with chemopotentiators, and programmable controlled drug release. Perspectives from materials and formulations at the nano-scales are specifically surveyed. Future opportunities and challenges are also discussed.

## Introduction

According to 2023 global statistics for cancer analysis [1], cancer recurrence and metastasis are the major causes of high mortality in patients clinically despite considerable achievements in cancer therapy. Chemotherapy is still the predominant form of systemic cancer treatment in clinics but suffers from drug resistance leading to cancer relapse and poor prognosis. Evidence has indicated a strong correlation between cancer stem-like cells (CSCs) and drug resistance [2, 3]. CSCs, also called tumor-initiating cells, which are characterized by self-renewal and tumorigenicity, have high resistance not only to traditional chemo- and radiotherapy but even to immunotherapy [4, 5]. With the development of cell biology, more and more drug resistance mechanisms of CSCs have been revealed. For example, CSCs upregulate the surface expression of adenosine triphosphate-binding cassette (ABC) efflux transporters to “pump” drugs outside the cells, leading to a low drug concentration within the cells [6]. Moreover, CSCs with powerful DNA repair abilities are highly tolerant to DNA damage treatments, which causes the failure of cell death regulation [7]. The content of CSCs shows a close association with high malignancy and poor prognosis [8–10]. Several cancer cell models are also used to explain why CSCs are malignant [11–13]. Compared with traditional multidrug resistance (MDR) models, CSCs tend to differentiate into varied tumor cells, resulting in a new heterogeneous and malignant microenvironment [14, 15], although both CSCs and MDR cells are insensitive to chemo- or radiotherapy and repopulate the tumors following treatment. Even worse, CSCs with higher malignancy have been identified to appear in a variety of cancers, including leukemia [16], breast [17], brain [18], colon [19], pancreatic [20], prostate [21], lung [22], liver [23], and skin cancers [24].

**Figure 1:**
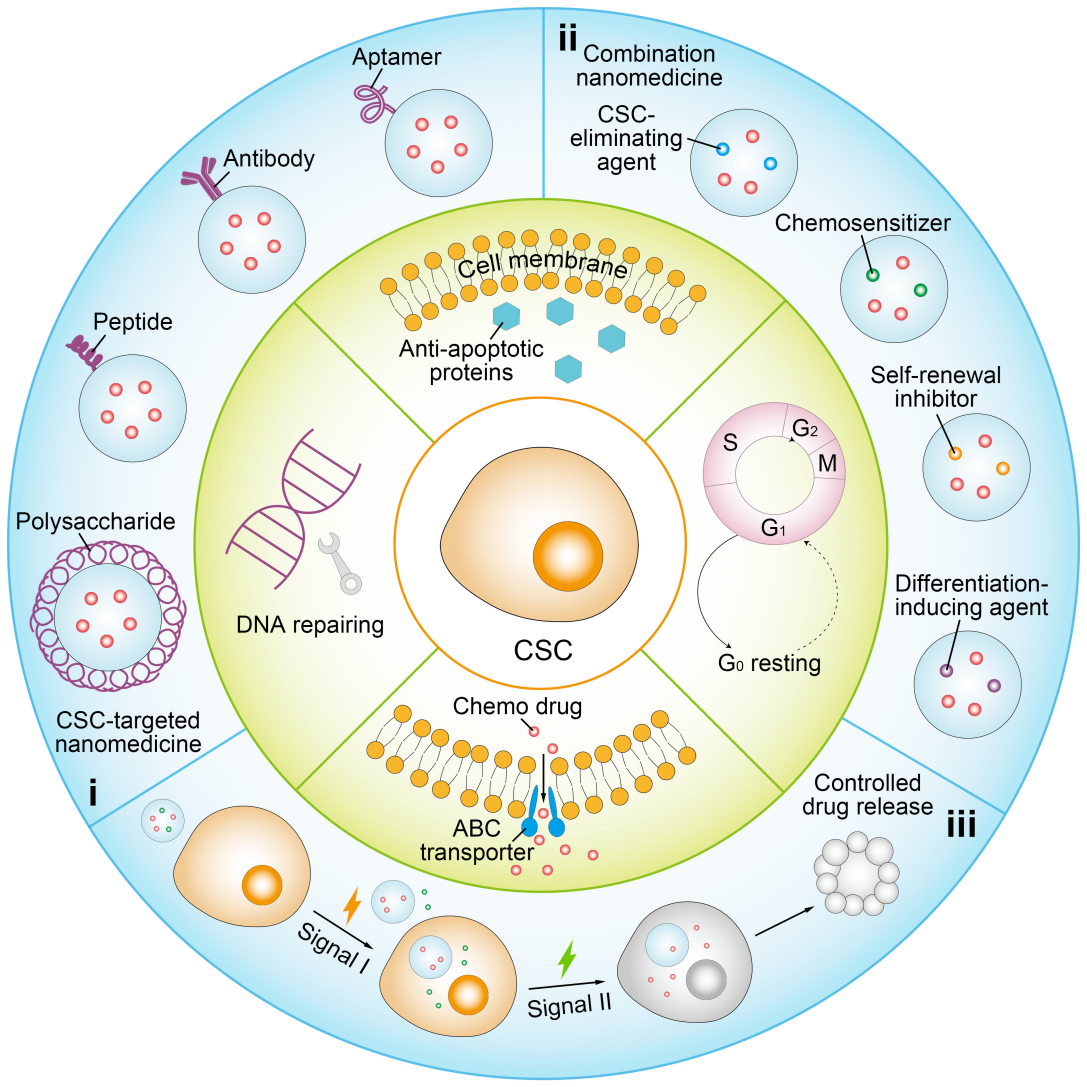
Schematic illustration of representative nanomedicine-based therapeutic strategies to overcome stemness-associated chemoresistance. i: CSC-targeted nanomedicine with ligand modification of polysaccharide, peptide, antibody or aptamer; ii: combination nanomedicine co-loaded with chemotherapeutic and chemopotentiator involving CSC-eliminating agent, chemosensitizer, self-renewal inhibitor, and differentiation-inducing agent; iii: controlled drug release in a programmed manner by responding to specific signals. S: DNA synthesis phase; M: mitotic phase; G_0_: resting phase

Nanomedicines provide great opportunities to treat CSCs. First of all, ligand conjugation on nanomedicines is a reasonable strategy to target CSCs as the expression of various biomarkers on the cellular surface [25], which not only enhances drug accumulation in CSCs but also protects normal stem cells from the toxicity of chemotherapeutics agents, even though CSCs share similar surface markers to normal stem cells. Secondly, the combination application of chemotherapeutic drugs with drug resistance reversing agents [26], self-renewal pathway inhibitors [27], and differentiation inducers [28] shows an enhanced effect on eradicating CSCs. More importantly, compared with free drugs, combinational drugs co-loaded in nanomedicines improve the efficiency to kill CSCs, because nanomedicines unify individual pharmacokinetic and biodistribution of combo drugs to achieve a fixed drug ratio [29, 30]. Finally, considering that combinatorial drugs have distinct anticancer targets and mechanisms, on-demand release of therapeutic agents acting on reversing resistance and chemotherapeutic drugs is important for enhancing their combination effects. Encouragingly, nanomedicines with programmable structures and engineered functions provide a platform for sustained and controlled drug release [31]. For example, layer-by-layer nanostructure allows the exteriorly-loaded drugs to be released faster than the interiorly-encapsulated drugs [32]. An alternative strategy is to physically encapsulate and chemically conjugate combination drugs in the nanocarriers, which supports sequential drug release dependent upon different release kinetics [33]. The chemical conjugation approach prevents premature drug release and increases nanoparticle (NP) stability, but also leads to inefficient drug release at the drug target site. Stimuli-triggered drug release strategy has widely been developed based on the tumor microenvironmental and cellular signals, such as hypoxia, acidity, and redox potential [34–36]. In this review, we summarize recent progress in nanomedicine-mediated therapeutic strategies to overcoming stemness-associated chemoresistance (Figure 1). Ligand-modified NPs targeting the specific biomarkers of CSCs and combination drugs co-loaded nanomedicine are surveyed. In particular, controlled drug release on potentiating synergistic drug effects is highlighted. The prospects of this field are also discussed.

## Nanomedicine targeting surface biomarkers of CSCs

The concept of ligand-receptor interaction is applied for the design of specific targeting nanomedicines [37] since the tumor tissues hold stark differences in expressions of specific receptors from normal tissues (receptor overexpression). However, the simple targeting strategies for bulk tumor cells are not efficient for CSCs. To this end, nanomedicines are engineered to target not only bulk tumor cells but also CSCs [38]. Spurred by the progression of cell and molecular biology, various surface biomarkers have been identified on the cell membranes of CSCs, which provide opportunities for nanomedicines to target both bulk tumor cells and CSCs. Among those biomarkers, the ligands commonly used for surface modification of nanomedicines are mainly aimed at the cell membrane receptors such as cluster of differentiation 44 (CD44), CD133, and CD20 [25, 39].

A host of ligands such as natural polysaccharides, peptides, antibodies, and aptamers have been employed for CSC-targeted drug delivery [40]. Hyaluronic acid (HA) is a natural polysaccharide that is found in the human body including skin, joints, and eyes, which has favorable biocompatibility and biodegradability, and particularly high binding affinity to the CD44 receptor [41–43]. The CD44 receptor has been discovered on hematopoietic stem cells and subsequently identified on CSCs of many types of solid tumors, such as pancreatic, breast, and prostate cancers [44]. Jafari Malek et al. [45] designed the *cis*-dichlorodiamminoplatinum (II) (CDDP)-HA NPs by agent-induced ionic gelation technique. CDDP, a positive agent containing amines was electrically interacted with carboxyl groups of HA to form the NPs. Compared with free CDDP, the CDDP-HA NPs significantly inhibited clonogenicity and tumorsphere formation and produced higher anti-proliferative activity on the CD44^+^ prostate (DU145 and PC3) cells *in vitro*. Kesharwani et al. [46] developed the HA-based micelles composed of a synthesized HA derivative that was obtained by conjugating copoly (styrene maleic acid) on the HA backbone. Curcumin (CUR), a hydrophobic small-molecular agent was physically encapsulated in the micelles. Elevation of cellular uptake of the micelles by the pancreatic CSCs [CD44^+^/CD133^+^/epithelial cell adhesion molecule^+^ (EpCAM^+^)] was determined compared with the non-CSC counterparts (CD44^–^/CD133^–^/EpCAM^–^), resulting in high cytotoxicity on the pancreatic CSCs. Treatment with the CUR-loaded micelles significantly decreased the CD44 expression and suppressed the nuclear factor-kappa B (NF-кB) signaling pathway, in turn leading to inhibition of proliferation and invasion.

**Figure 2:**
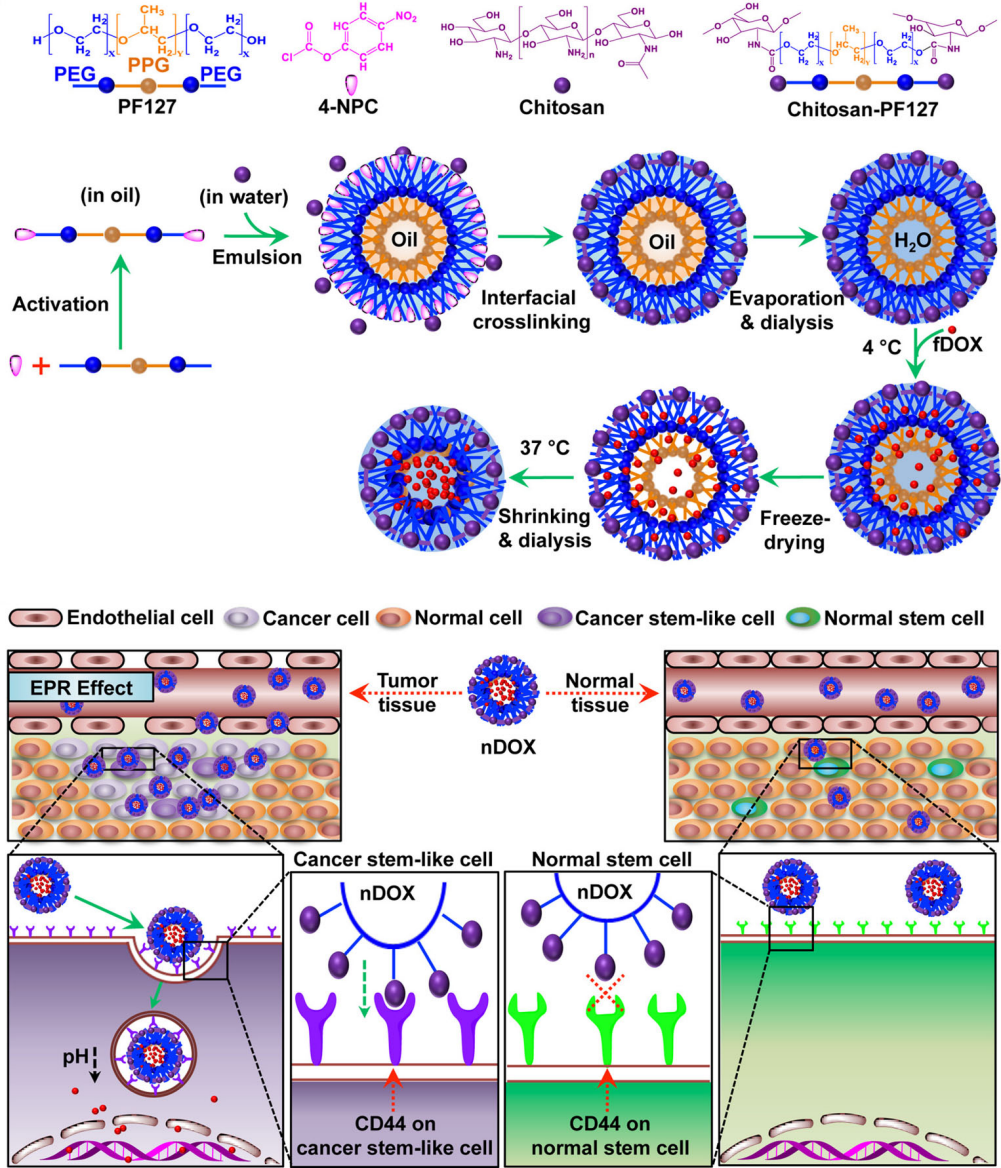
CS-modified polymeric micelles (PMs) for CSC-targeted delivery of DOX. PF127: pluronic F127; 4-NPC: 4-nitrophenyl chloroformate; H_2_O: hydrogen oxide; EPR: enhanced permeability and retention effect; fDOX: free DOX; nDOX: nano DOX *Note.* Reprinted with permission from “Chitosan-decorated doxorubicin-encapsulated nanoparticle targets and eliminates tumor reinitiating cancer stem-like cells,” by Rao W, Wang H, Han J, Zhao S, Dumbleton J, Agarwal P, et al. ACS Nano. 2015;9:5725–40 (https://pubs.acs.org/doi/10.1021/nn506928p). © 2015 American Chemical Society.

Chitosan (CS), a polysaccharide that is derived from the shells of crustaceans, such as shrimps, lobsters, and crabs, also presents a high CD44-binding affinity. Rao et al. [47] prepared CS-modified poly(ethylene glycol) (PEG)-poly(propylene glycol) (PPG)-PEG micelles using an emulsion method for eradicating CD44-overexpressing breast CSCs (Figure 2). CS was interfacially crosslinked on the surface of the micelles, and doxorubicin (DOX) was loaded in the micelles. The micelle-mediated delivery increased the cytotoxicity of DOX on the CD44^+^ breast cancer cells (MCF-7) by six times compared with the application of free DOX solution. The DOX-loaded micelles showed a superior effect on inhibiting the tumor growth than the free DOX in an orthotopic CSC-enriched breast tumor mouse model but did not cause any noticeable systemic toxicity. Kuo et al. [48] reported the poly(lactic-co-glycolic acid) (PLGA)/CS-based NPs modified with sialic acid to permeate the blood-brain barrier (BBB) and with anti-aldehyde dehydrogenase (ALDH) antibody to target glioblastoma CSCs. CUR was embedded in the hydrophobic core of the PLGA. The NPs enhanced the transportation across the BBB-mimic cell monolayered model established by human brain microvascular endothelial cells, human astrocytes, and human brain vascular pericytes. In addition, the increase in cell internalization was determined after treatment with the NPs, resulting in a high level of cytotoxicity against ALDH^+^ U87MG CSCs.

**Figure 3:**
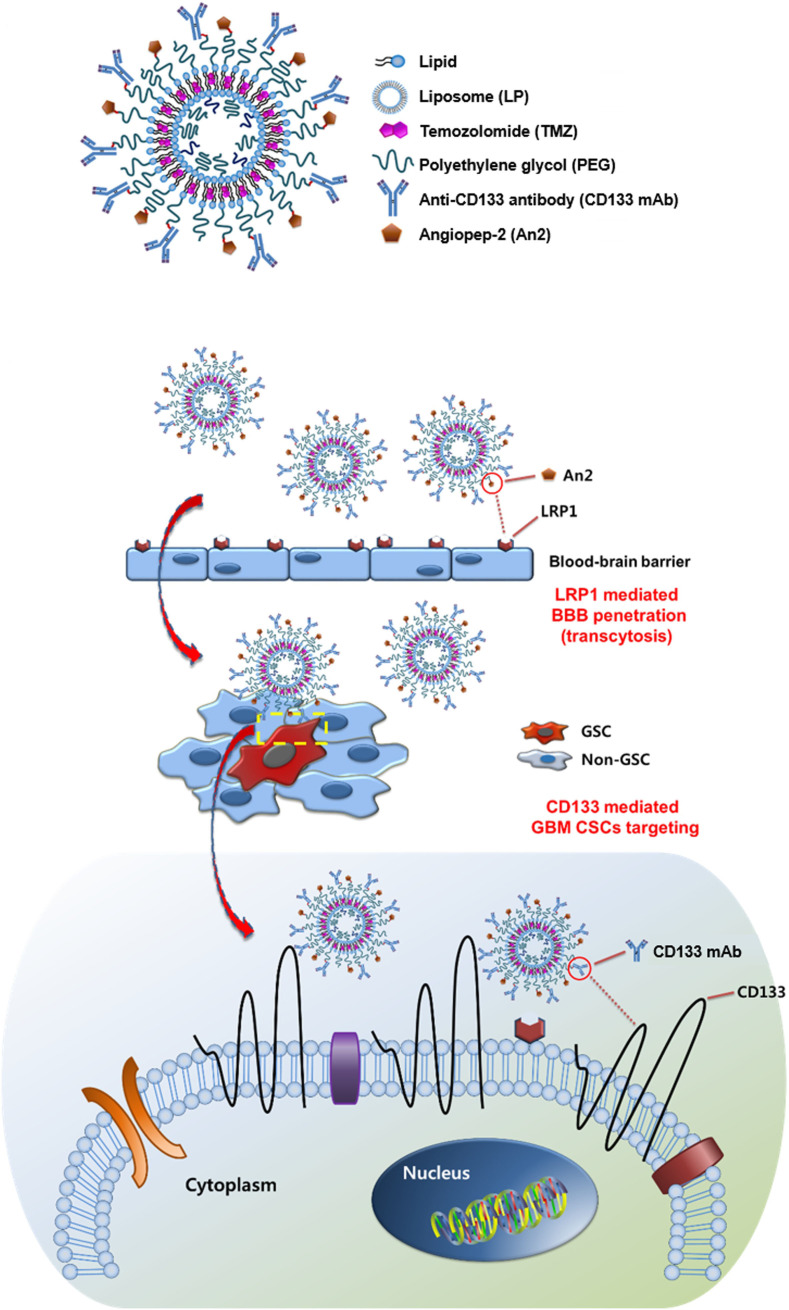
Immunoliposomes modified with angiopep-2 and anti-CD133 antibody for CSC-targeted delivery of TMZ. LRP1: lipoprotein receptor-related protein 1; GCS: glioma stem cell *Note.* Reprinted with permission from “Dual-targeting immunoliposomes using angiopep-2 and CD133 antibody for glioblastoma stem cells,” by Kim JS, Shin DH, Kim JS. J Control Release. 2018;269:245–57 (https://www.sciencedirect.com/science/article/abs/pii/S0168365917310222?via%3Dihub). © 2017 Elsevier B.V.

In addition to natural polysaccharides, biomolecules such as peptides and antibodies with high and specific binding efficiency to the receptors have also been modified on the nanocarriers to target CSCs. Cho et al. [49] screened a small peptide, named CBP4 using a phage display technique, and validated highly binding to CD133 which is a biomarker for glioblastoma CSCs. The CBP4 peptide was conjugated on gold NPs, which possessed fluorescent signal “on-off” properties in the presence of the target and was used as an imaging agent for the diagnosis of glioblastoma. Kim et al. [50] formulated an immunoliposome modified with angiopep-2 and anti-CD133 antibody to overcome BBB and enhance specific delivery of temozolomide (TMZ) to glioblastoma CSCs (Figure 3). The dual-targeting immunoliposome increased the cytotoxicity of TMZ against CD133^+^/ALDH1^+^ U87MG-TL CSCs by 425 times and 181 times compared with free drug and non-targeted plain liposome (LP), respectively, and significantly augmented migration inhibition and apoptosis induction. Elevation of BBB penetration and glioblastoma targeting was accomplished by the immunoliposome. Treatment with the TMZ-loaded immunoliposome inhibited tumor growth and prolonged survival in orthotopic glioblastoma-bearing mice. Chen et al. [51] developed PLGA-based NPs containing 1,2-distearoyl-sn-glycero-3-phosphoethanolamine (DSPE)-PEG-maleimide for surface modification of both anti-CD133 and anti-CD44 antibodies. All-*trans* retinoic acid (ATRA), a differentiation-inducing agent was physically encapsulated in the PLGA NPs. Compared with either non-modified or mono-modified NPs, the dual-modified NPs significantly increased the *in vitro* cytotoxicity of ATRA toward the CD44^+^/CD133^+^ gastric (MKN-45 and NCI-N87) CSCs.

**Figure 4:**
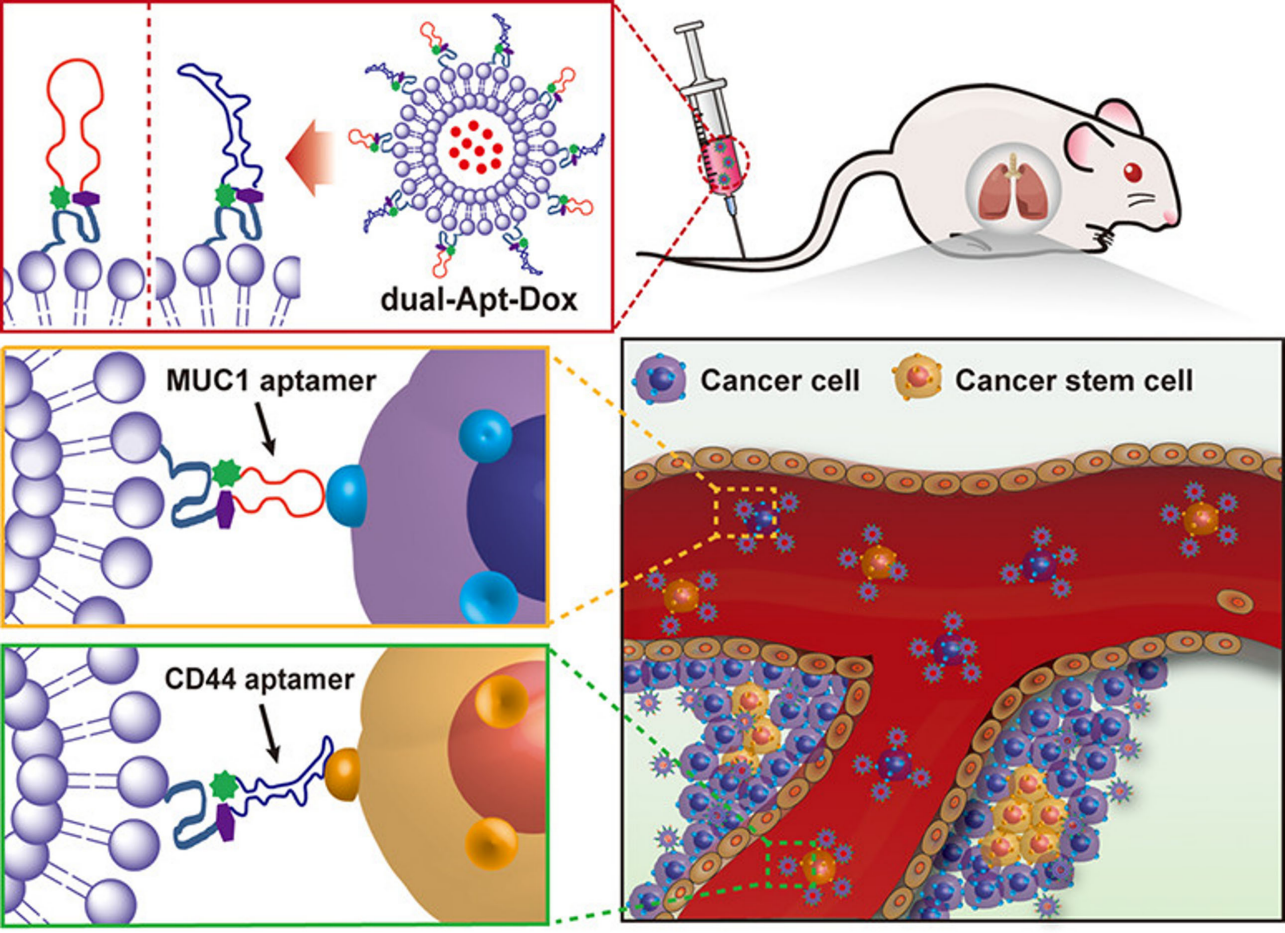
Liposomes modified with CD44 and MUC1 aptamers for respective targeted delivery of DOX to breast CSCs and cancer cells. dual-Apt-Dox: dual-aptamosomes harboring DOX *Note.* Reprinted with permission from “Anti-MUC1/CD44 dual-aptamer-conjugated liposomes for cotargeting breast cancer cells and cancer stem cells,” by Kim DM, Kim M, Park HB, Kim KS, Kim DE. ACS Appl Bio Mater. 2019;2:4622–33. (https://pubs.acs.org/doi/10.1021/acsabm.9b00705). © 2019 American Chemical Society.

Aptamers are short-stranded nucleotide sequences, usually DNA or RNA of 20 to 100 nucleotides. Due unique tertiary structure, the aptamers can identify target molecules through their three-dimensional conformation with a high binding affinity [52, 53]. The aptamers have many advantages over antibodies, such as easy acquirability by chemical synthesis, low cost, high stability, and strong specificity. Zeng et al. [54] designed salinomycin (Sal)-loaded CD20 aptamer-modified NPs by nanoprecipitation method to target CD20^+^ melanoma CSCs. The CD20 aptamer was decorated on the NPs via a maleimide-thiol reaction. The uptake of CD20-modified NPs significantly increased compared with that of either unmodified NPs or free Sal. The CD20-modified NPs showed strong anticancer efficacy by eliminating the CD20^+^ melanoma CSCs both *in vitro* and *in vivo*. Kim et al. [55] reported dual-aptamer-decorated liposomes (called dual-aptamosomes) for respective targeted delivery of DOX to breast CSCs and cancer cells (Figure 4). Two types of DNA aptamers were used: one was a CD44 aptamer for targeting CD44^+^ breast CSCs, and the other was an aptamer for targeting transmembrane glycoprotein mucin 1 (MUC1) expressed on breast cancer cells. The DOX-loaded dual-aptamosomes showed significantly higher cytotoxicity against both breast CSCs and cancer cells compared with the DOX-loaded liposomes, and suppress the metastasis of breast CSCs in the mouse models. Recently, Yin et al. [56] developed an aptamer-drug conjugate of CD133 aptamer and DOX for targeted delivery to liver CSCs. The CD133 aptamer-DOX conjugate promoted the accumulation of DOX within the CD133^+^ liver (Huh7 and PLC/PRF/5) CSCs and significantly suppressed the tumorsphere formation compared with the free DOX. Further *in vivo*, investigation of the CD133 aptamer-DOX conjugate for targeted drug delivery and therapeutic efficacy should be performed to demonstrate its potential for treatment of liver cancer.

## Nanomedicine for co-delivery of chemopotentiator and chemotherapeutic

Nanomedicines modified ligands for specific surface biomarkers of CSCs play an essential role in elevating the intracellular concentration of chemotherapeutic drugs. However, the efficacies of chemotherapeutic drugs are still limited because CSCs harbor multiple chemoresistant mechanisms causing the treatment failure of single or combinational chemotherapeutics [57]. For example, conventional chemotherapeutic drugs are effective to kill fast-growing cancer cells, but not efficient on the mitotically quiescent CSCs [58]. The overexpressed ABC transporters on CSCs pump the intracellularly released drugs outside the cells, leading to drug concentration reduction within the cells [59]. CSCs also upregulated expressions of several anti-apoptotic proteins [60], and have strong DNA-repair and reactive oxygen species (ROS)-scavenging capacities [61, 62]. Moreover, bulk tumor cells may undergo dedifferentiation to regenerate CSCs under the tumor microenvironment [63]. Accordingly, the combined application of chemo potentiators to sensitize CSCs and overcome chemoresistance in different ways provides opportunities for enhanced chemotherapy on stemness-derived resistant tumors (Table 1).

**Table 1:** Representative combination nanomedicines to overcome stemness-associated chemoresistance

**Chemopotentiator**		**Chemotherapeutic drug**	**Nanocarrier**	**Cancer (cell line)**	**Reference**
CSC-eliminating agent	Sal	PTX	Micelle	Breast cancer (MCF-7)	[67]
	Sal	SN38	NP	Colon cancer (HCT-116)	[68]
	Sal	DOX	LP	Liver cancer (Huh-7)	[70]
	THZ	PTX	LP	Breast cancer (MCF-7)	[104]
Chemosensitizer	Tar	DOX	MSN	Breast cancer (MDA-MB-231)	[75]
	siABCG2 and siBCL-2	DOX	Noisome	Breast cancer (MDA-MB-231)	[80]
	CUR	PTX	NP	Breast cancer (MCF-7)	[82]
	RUB	DTX	Micelle	Prostate cancer (PC3)	[106]
Self-renewal inhibitor	siNotch1	Pt	Micelle	Liver cancer (SMMC7721)	[86]
	siBmi1	UA	LP	Epidermoid cancer (KB)	[87]
	Wnt and uPAR peptides	DOX	NP	Breast cancer (MDA-MB-231)	[88]
Differentiation-inducing agent	ATRA	DOX	NP	Breast cancer (MDA-MB-231)	[94]
	ATRA	DTX	Dendrisome	Breast cancer (MCF-7 and SK-BR-3)	[95]
	ATRA	IRI	NP	Breast cancer (4T1)	[96]
	ATRA	CPT	NP	Breast cancer (MCF-7)	[110]
	ATRA	DOX	NP	Breast cancer (4T1)	[111]

Tar: tariquidar; siABCG2: small interfering RNA (siRNA) targeting ABC subfamily G member 2; siBCL-2: siRNA targeting B-cell lymphoma-2; siNotch1: siRNA targeting Notch receptor 1; siBmi1: siRNA targeting BMI1 proto-oncogene, polycomb ring finger; uPAR: urokinase plasminogen activator receptor; PTX: paclitaxel; IRI: irinotecan; CPT: camptothecin; MSN: mesoporous silica NP; RUB: rubone; UA: ursolic acid; Pt: platinum; THZ: thioridazine; SN38: 7-ethyl-10-hydroxycamptothecin; Wnt: Wnt/β-catenin signaling

## Co-delivery of CSC-eliminating agent and chemotherapeutic drug

Multiple signaling pathways of CSCs are changed in comparison with that of normal cancer cells, which renders CSCs to be more resistant to traditional chemotherapeutic drugs. The discovery and development of specific CSC-eliminating agents is a research hotspot in the field of combating malignancy [64]. However, the activity of these agents on normal cancer cells is relatively limited, and thus the combination of traditional chemotherapy drugs and CSC-eliminating agents is expected to be a promising approach to killing the bulk tumor cells and CSCs together for reversing stemness-associated chemoresistance [65].

Sal, an ionophore antibiotic has been found to selectively eliminate the CSC-like population of solid tumors compared with conventional chemotherapeutic drugs including PTX [65, 66]. However, unacceptable cardiotoxicity and neurotoxicity limit the clinical translation and application of Sal. Nanocarriers have been increasingly explored for delivery of Sal to improve its biodistribution and efficacy, and to reduce the toxicities. Zhang et al. [67] utilized Sal-loaded PEG-block-poly(-caprolactone) (PEG-*b*-PCL) micelles (M-Sal) to increase the therapeutic efficacy of octreotide-modified PTX (Oct-M-PTX)-loaded PEG-*b*-PCL micelles on breast cancers. M-Sal presented significantly higher activity than free Sal to reduce the cell population with the CD44^+^/CD24^–^ phenotype on the breast cancer cells (MCF-7) and the mouse tumor model. The combined application of M-Sal enhanced the *in vivo* antitumor efficacy of both taxols (the commercial PTX preparation) and Oct-M-PTX by eliminating the breast CSCs and cancer cells. Tsakiris et al. [68] prepared the lipid NPs for the respective delivery of Sal and SN38 (the active form of IRI) to eradicate both proliferating colon cancer cells and resistant colon CSCs. Sal and SN38 acted on the colon (HCT116) CSCs and cancer cells, respectively. The Sal-loaded lipid NPs significantly reduced the toxicity of Sal including hemolysis, but promoted the Sal-mediated suppression of clonogenicity and tumorsphere formation compared with free Sal. Co-treatment with Sal-loaded and SN38-loaded lipid NPs resulted in enhanced tumor growth inhibition and prolonged survival in the mouse model of the HCT116 tumor.

**Figure 5:**
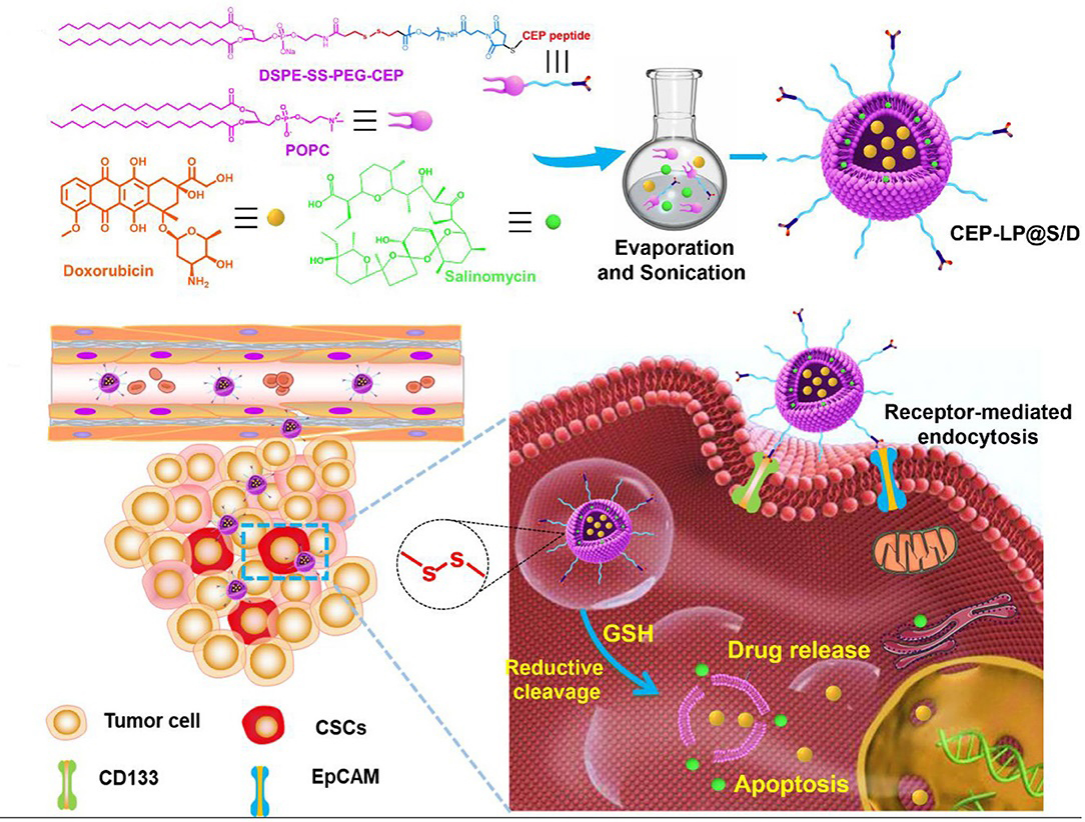
LPs modified with Y-shaped ligand by integration of CD133- and EpCAM-targeted peptides for co-delivery of Sal and DOX to liver CSCs. SS: disulfide linker; POPC: 1-palmitoyl-2-oleoyl-sn-glycero-3-phosphorylcholine; GSH: glutathione *Note.* Reprinted from “A novel CD33- and EpCAM-targeted liposome with redox-responsive properties capable of synergistically eliminating liver cancer stem cells,” by Wang Z, Sun M, Li W, Fan L, Zhou Y, Hu Z. Front Chem. 2020;8:649 (https://www.frontiersin.org/articles/10.3389/fchem.2020.00649/full). CC BY.

Co-encapsulation of combination drugs in one nanocarrier plays a crucial role in improving pharmacokinetic profiles and maintaining drug ratio, which markedly augments therapeutic efficacy. Gao et al. [69] reported rigid polymeric NPs composed of PLGA and *D*-α-tocopherol polyethylene glycol succinate (TPGS) for co-delivery of Sal and docetaxel (DTX) to unify their pharmacokinetics and preserve a fixed synergic ratio. The Sal/DTX co-loaded PLGA/TPGS NPs could prolong the circulation time of two drugs and maintain the synergistic drug ratio for 24 h, resulting in a superior effect on suppressing tumor growth on the breast (MCF-7) tumor mouse models than a mixture of Sal-loaded and DTX-loaded NPs. Wang et al. [70] developed dual-targeted redox-responsive LPs co-loaded with Sal and DOX for the treatment of stemness-high liver cancer (Figure 5). A synthesized lipid containing a Y-shaped ligand by integration of CD133- and EpCAM-targeted peptides and a redox-degradable disulfide linker was incorporated in the LP components. The obtained LPs supported selective targeting of CD133^+^ EpCAM^+^ liver CSCs by the peptide ligand and promoted intracellular drug release by the LP destruction due to the degradation of the synthesized lipid under the reductive cytoplasmic environment with a high level of glutathione. The combination of DOX-induced cell apoptosis and Sal-mediated lysosomal iron sequestration generated an efficient effect on simultaneously eliminating liver CSC and bulk tumor cells, yielding increased anticancer efficacy on the mouse model of the CSC-enriched liver tumor.

In addition to producing a direct effect on specifically killing the CSCs, Sal was also able to induce upregulation of expressions of certain surface receptors on the CSCs to potentiate the efficacy of corresponding targeting therapeutics [71]. Antibody-based therapeutics have achieved great success in clinical cancer treatment, which bind to specific receptors overexpressed on cancer cells and induce cell apoptosis, such as cetuximab/panitumumab [targeting epidermal growth factor receptor (EGFR)], trastuzumab [targeting human EGFR 2 (HER2)] and conatumumab/lexatumumab/tumor necrosis factor-related apoptosis-inducing ligand [TRAIL, targeting death receptor 5 (DR5)]. However, compared with bulk tumor cells, CSCs show low expressions of receptors, causing limited effect of antibody-mediated cancer treatment. For example, lower expression of DR5 on the colon CSCs has been demonstrated as one of the key factors leading to the high resistance of colon cancer to TRAIL [72]. Shen et al. [73] recently constructed the HA-coated LPs for the co-delivery of plasmid DNA encoding TRAIL (pTRAIL) and Sal to overcome the stemness-derived TRAIL resistance of colon cancer. Equipped with HA, the LP exhibited high accumulation in the CSC-enriched colon tumor with overexpression of the CD44 receptor. The pTRAIL/Sal co-loaded LPs rendered the colon cancer cells to express TRAIL and sensitize the colon CSCs by Sal-induced upregulation of DR expression to TRAIL-mediated apoptosis, which showed a high capacity to inhibit tumor growth in the orthotopic CSC-enriched colon tumor mouse model.

## Co-delivery of chemosensitizer and chemotherapeutic drug

**Figure 6:**
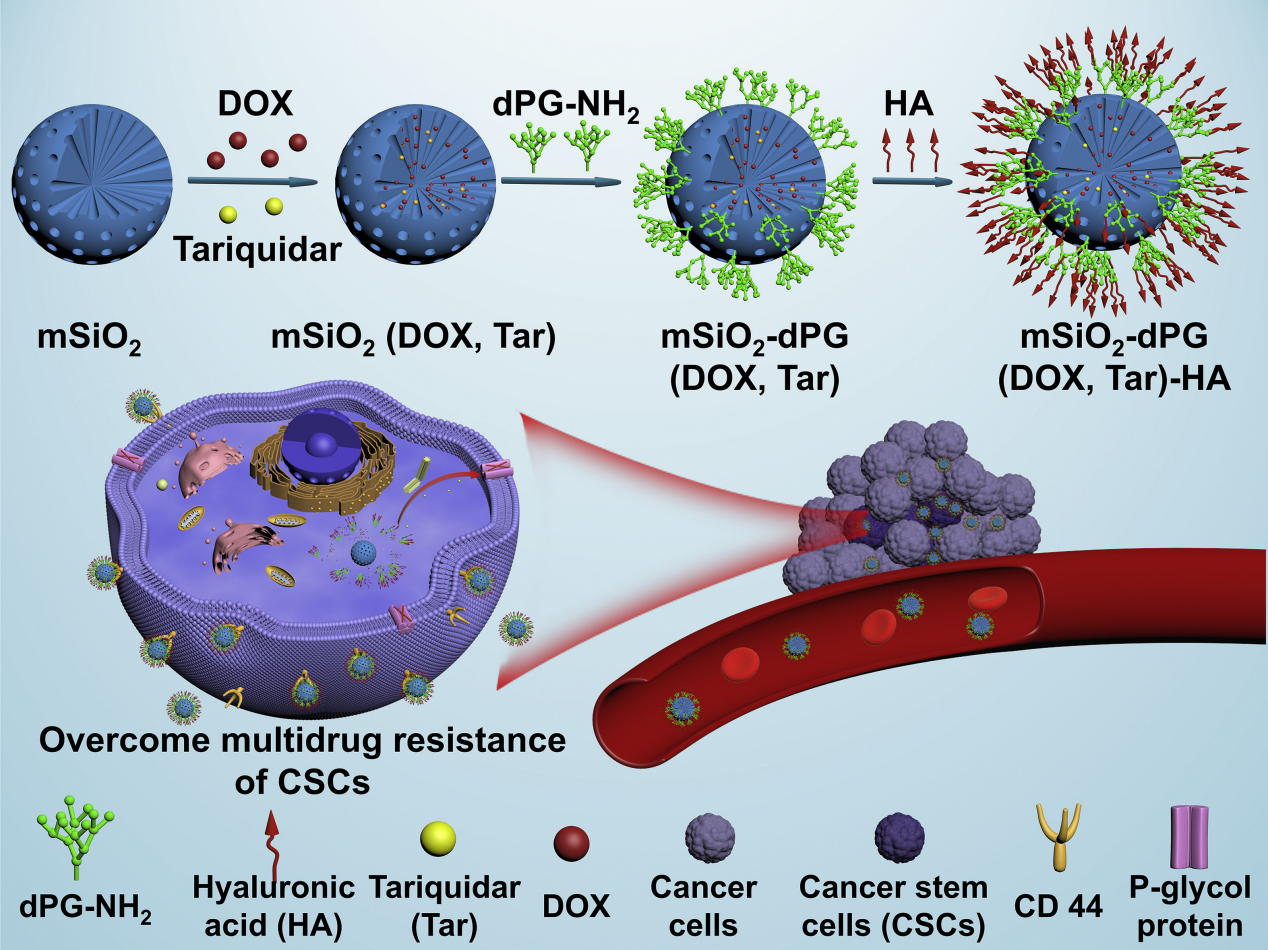
HA-coated MSNs for co-delivery of Tar and DOX to overcome chemoresistance by inhibiting P-gp-mediated drug efflux of breast CSCs. mSiO_2_: mesoporous silica *Note.* Reprinted with permission from “Novel dendritic polyglycerol-conjugated, mesoporous silica-based targeting nanocarriers for co-delivery of doxorubicin and tariquidar to overcome multidrug resistance in breast cancer stem cells,” by Pan Y, Zhou S, Li Y, Parshad B, Li W, Haag R. J Control Release. 2021;330:1106–17. (https://www.sciencedirect.com/science/article/abs/pii/S0168365920306647?via%3Dihub). © 2021 Elsevier B.V.

P-glycoprotein (P-gp) is a typical class of ABC efflux transporters with broad specificity [74]. Many clinically-applied chemotherapeutic drugs, including anthracyclines, vinca alkaloids, and taxanes are the substrates of P-gp, which actively transports the drugs outside the cells, leading to a reduction in the intracellular drug concentration. High expression of P-gp has been identified to be one of the major features in the population of CSCs [14]. Of note, chemotherapy also induces elevation of the P-gp expression, causing exacerbation of chemoresistance. Pan et al. [75] reported the HA-coated MSNs with amine-modified dendritic polyglycerol (dPG) as a pH-responsive “gatekeeper” for the co-delivery of DOX and Tar to combat the P-gp-mediated chemoresistance of breast CSCs (Figure 6). Tar is a potent and specific inhibitor of P-gp with high affinity [76], which is being investigated in combination with chemotherapeutic drugs such as DOX and DTX for the treatment of many cancers in clinical trials. The HA-based CD44 receptor targeting and Tar-mediated P-gp inhibition of MSNs promoted the elevation of DOX accumulation with the ALDH^+^ MDA-MB-231 CSCs, and the Schiff base bonds with pH-triggered degradation property accelerated the release of DOX from MSNs in the acidic tumor microenvironment. Treatment with Tar/DOX co-loaded MSNs reduced expressions of typical stemness-associated transcription factors including SRY-box transcription factor 2 (Sox2), octamer-binding transcription factor 4 (Oct4), and Nanog homeobox (Nanog), resulting in enhanced inhibition of tumorsphere formation of the MDA-MB-231 CSCs *in vitro*.

In addition to the ABC transporter-mediated drug efflux leading to intracellular drug concentration decrease, upregulated expression of anti-apoptotic proteins, such as B-cell lymphoma-2 (BCL-2) [77], survivin [60], and X-linked inhibitor of apoptosis (XIAP) [78], is also one of an important contributor to therapeutic resistance. BCL-2 is a widely-investigated anti-apoptotic protein located mainly within the outer membrane of the mitochondrion, which has been found to be highly expressed in many types of CSCs to counteract the apoptotic death induced by chemotherapeutics [79]. Sun et al. [80] developed the cationic niosomes consisting of 1,2-dioleoyl-3-trimethylammonium-propane (DOTAP), *D*-α-TPGS and sorbitan monooleate 80 (Span 80) for co-delivery of DOX with siABCG2 and siBCL-2 to reverse resistance of breast CSCs to DOX. Delivered by the niosomes, siABCG2 downregulating the ABC subfamily G member 2 (ABCG2) expression increased the concentration of DOX within the CD44^+^/CD24^–^ MDA-MB-231 CSCs and siBCL-2 reducing the BCL-2 expression boosted the DOX-triggered apoptosis of the CSCs. The siABCG2/BCL-2/DOX co-loaded noisomes effectively sensitized the MDA-MB-231 CSCs to DOX and showed superior *in vitro* cytotoxicity than DOX-loaded, DOX/siABCG2 co-loaded or DOX/siBCL-2 co-loaded niosomes.

Epithelial-to-mesenchymal transition (EMT) is a biological process whereby polarized epithelial cells transform into mesenchymal cells, which participate in the development processes of cancer associated with drug resistance, stemness maintenance, cancer invasiveness, and metastasis [81]. It has been found that the cancer cells undergoing EMT acquired stem-like properties, and these undifferentiated mesenchymal upregulated expression of stemness-associated transcription factors such as Oct4 and Nanog [2]. Yang et al. [82] fabricated HA-coated PLGA NPs for the co-delivery of PTX and CUR to inhibit EMT and increase the anticancer efficacy of PTX to the breast CSCs. The anticancer mechanism of CUR has been evidenced to be associated with the suppression of EMT. The NPs targeted the MCF-7 CSCs through the HA-CD44 receptor interaction, decreased the CSC population with mammosphere formation, and inhibited the migration of CSCs as validated by upregulated expression of E-cadherin and downregulated expression of β-catenin. On the MCF-7 tumor-bearing mice, treatment with the PTX/CUR co-loaded NPs reinforced anticancer efficacy by inhibiting the growth of both breast CSCs and bulk tumor cells. Zhao et al. [83] developed PEG-PLGA NPs conjugated by HA on the surface to simultaneously deliver Sal and CUR. The HA modification elevated the cellular uptake of the NPs by the CD44^+^/CD24^–^ MCF-7 CSCs. The Sal/CUR co-loaded NPs showed a high effect on inducing G1 cell cycle arrest and suppressing cell migration and proliferation of the MCF-7 CSCs *in vitro*.

## Co-delivery of self-renewal signaling pathway inhibitor and chemotherapeutic drug

**Figure 7:**
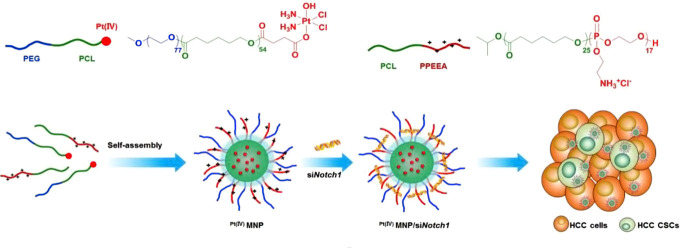
Polymeric micelleplexs for co-delivery of siNotch1 and Pt to overcome chemoresistance by inhibiting the Notch1-related self-renewal signaling pathway of liver CSCs. PPEEA: poly(2-aminoethyl ethylene phosphate); MNP: micellar NP; HCC: hepatocellular carcinoma; CI: chloride ion *Note.* Reprinted with permission from “Co-delivery of platinum drug and *siNotch1* with micelleplex for enhanced hepatocellular carcinoma therapy,” by Shen S, Sun CY, Du XJ, Li HJ, Liu Y, Xia JX, et al. Biomaterials. 2015;70:71–83. (https://linkinghub.elsevier.com/retrieve/pii/S0142-9612(15)00692-4). © 2015 Elsevier B.V.

Studies have found that stem-related signaling pathways such as Notch, Wnt, and hedgehog are essential for CSCs to maintain their stemness, and CSCs lose their ability to self-renew and differentiate as the relevant signaling pathways are inhibited [84, 85]. Therefore, stem-related signaling pathway inhibitors can transform drug-resistant CSCs into sensitive common tumor cells, and the combination with chemotherapy drugs can effectively improve the therapeutic effect of chemotherapy [27]. Shen et al. [86] reported PMs-mediated co-delivery of Pt and siNotch1 for the treatment of CSC-harboring hepatocellular carcinoma (Figure 7). Pt and siNotch1 were delivered by the micelles into both liver CSCs and cancer cells. The Pt/siNotch1 co-encapsulated micelles efficiently down-regulated expression of the Notch receptor 1 (Notch1) signaling pathway and increased the response of the liver CSCs to Pt, leading to enhancement of proliferation inhibition and apoptosis induction. Elevation of tumor growth inhibition was determined by the micelle-mediated CSC elimination in the SMMC7721 tumor mouse model. Upregulation of the BMI1 proto-oncogene, polycomb ring finger (*Bmi1*) gene is one of the characteristic features of CSCs connected to self-renewal and stemness maintenance in a variety of solid tumors. Li et al. [87] constructed folate-decorated cationic LPs co-loaded with siBmi1 and UA for enhanced anticancer efficacy. UA is a natural product that has been validated to have biological functions, such as anti-cancer, anti-oxidative, and anti-inflammation activities. The targeted LPs increased the cellular uptake by the oral epidermoid carcinoma (KB) cells with overexpression of folate receptors. After treatment with the siBmi1/UA co-loaded LPs, the *Bmi1* gene and protein expressions were reduced, and increased cytotoxicity against the KB cells was found. Moreover, the tumor-bearing mice receiving the siBmi1/UA co-loaded LPs showed significantly higher tumor-inhibitory effects than either siBmil-loaded or UA-loaded LPs. Alternatively, Miller-Kleinhenz et al. [88] developed ultra-small magnetic iron oxide NPs (IONPs) that were conjugated with two types of peptide ligands dually targeting the Wnt receptor, LRP5/6, and uPAR for delivery of DOX and treatment of resistant breast cancer. The dual-targeted IONPs efficiently bond to both LRP5/6 and uPAR, resulting in inhibition of the Wnt/β-catenin signaling and the CSC-like phenotype of cancer cells as validated by a noticeable reduction in the expressions of LRP5/6, CD44, and uPAR. The DOX-loaded dual-targeted IONPs had a stronger effect on suppressing the tumor growth than the DOX-loaded single-targeted IONPs in the human breast tumor PDX model. Yang et al. [89] developed micelles composed of two polymer-based prodrugs that were synthesized by cyclopamine (CYP) and PTX respectively conjugated on an amphiphilic polymer PEG-block-poly(2-methyl-2-carboxyl-propylene carbonate-graft dodecanol). The combination of CYP-mediated blockade of the Hedgehog signaling pathway and PTX-induced cell apoptosis significantly inhibited the colony formation of the PTX-resistant prostate (DU145-TXR and PC3-TXR) CSCs. Treatment with CYP/PTX co-loaded micelles resulted in the upregulation of tumor suppressors microRNAs (miRNAs) and inhibition of the hedgehog signaling pathway.

## Co-delivery of differentiation-inducing agent and chemotherapeutic drug

Differentiation therapy is a potent anticancer approach in clinics to promote malignant cells to differentiate into more mature phenotypes by using differentiation-inducing agents. The great success is the application of ATRA and arsenic trioxide for the treatment of patients with acute promyelocytic leukemia, resulting in nearly complete remission with cure rates over 90% [90]. Accumulating evidence has indicated that ATRA is also able to induce differentiation of the CSCs in many solid tumors, and therefore potentiate the cytotoxic effects of chemotherapeutic drugs on the CSCs [91–93]. Sun et al. [94] reported ATRA/DOX co-loaded PLGA NPs (ATRA/DOX-NPs) for eliminating both bulk tumor cells and CSCs. The NPs promoted the concentrations of both ATRA and DOX within the ALDH^+^ MDA-MB-231 CSCs compared with free drugs. ATRA/DOX-NPs efficiently induced differentiation of the MDA-MB-231 CSCs as presented by a marked reduction in the expressions of the stemness-associated transcription factors, Nanog, Sox2, and Oct4, leading to a significant decrease of the CSC population and self-renewal capacity. Moreover, the co-delivery by NPs not only prolonged the circulation time but also increased the tumor accumulation of both drugs. Treatment with ATRA/DOX-NPs significantly enhanced tumor growth inhibition with reduced frequency of the CSCs on the orthotropic breast tumor mouse model compared with other treatments including a mixture of ATRA-NPs and DOX-NPs.

To elevate the tumor and cell penetration, Mu et al. [95] developed ATRA/DTX co-encapsulated dendrisomes composed of amphiphilic dendrimers and cell-penetrating peptide-conjugated lipids for a combination of differentiation therapy and chemotherapy. The dendrisomes exhibited increased cellular uptake and lysosomal escape on the breast (MCF-7 and SK-BR-3) CSCs, promoted penetration into the tumorpshere, and elevated tumor accumulation of the tumor-bearing mice. Treatment with the ATRA/DTX co-loaded dendrisomes resulted in differentiation and sensitization of CSCs to DOX, yielding a significant reduction of the CD44^+^ CD24^–^ CSC-like population. Upon differentiation, signal molecules [retinoic acid receptor α (RARα), calmodulin 3 (CALM3), cyclin dependent kinase (CDK) inhibitor 1A (CDKN1A), CDK7, NDC1 transmembrane nucleoporin (NDC1), and bone morphogenetic protein 2 (BMP2)], transcription factors [POU class 5 homeobox 1 (POU5F1), oligodendrocyte transcription factor 3 (OLIG3), twist family BHLH transcription factor 2 (TWIST2), and Snail family transcriptional repressor 1 (Snail1)], and surface markers [forkhead box O3 (FoxO3)] were upregulated, while signal molecules [CDK2, cyclin E1 (CCNE), CCCTC-binding factor like (CTCFL), inhibitor of DNA binding 3 (ID3), and methionine adenosyltransferase 1A (MAT1A)], transcription factors [Sox15, Sox-6, POU2F1, KLF transcription factor 5 (KLF5), and Nanog], and surface markers [CD44, C-X-C motif chemokine receptor 4 (CXCR4), nestin (NES), ALDH 4 family member A1 (ALDH4A1), and ALDH1L2] were downregulated. The tumor-bearing mice treated with the ATRA/DTX co-loaded dendrites presented high tumor inhibition and apoptosis induction.

**Figure 8:**
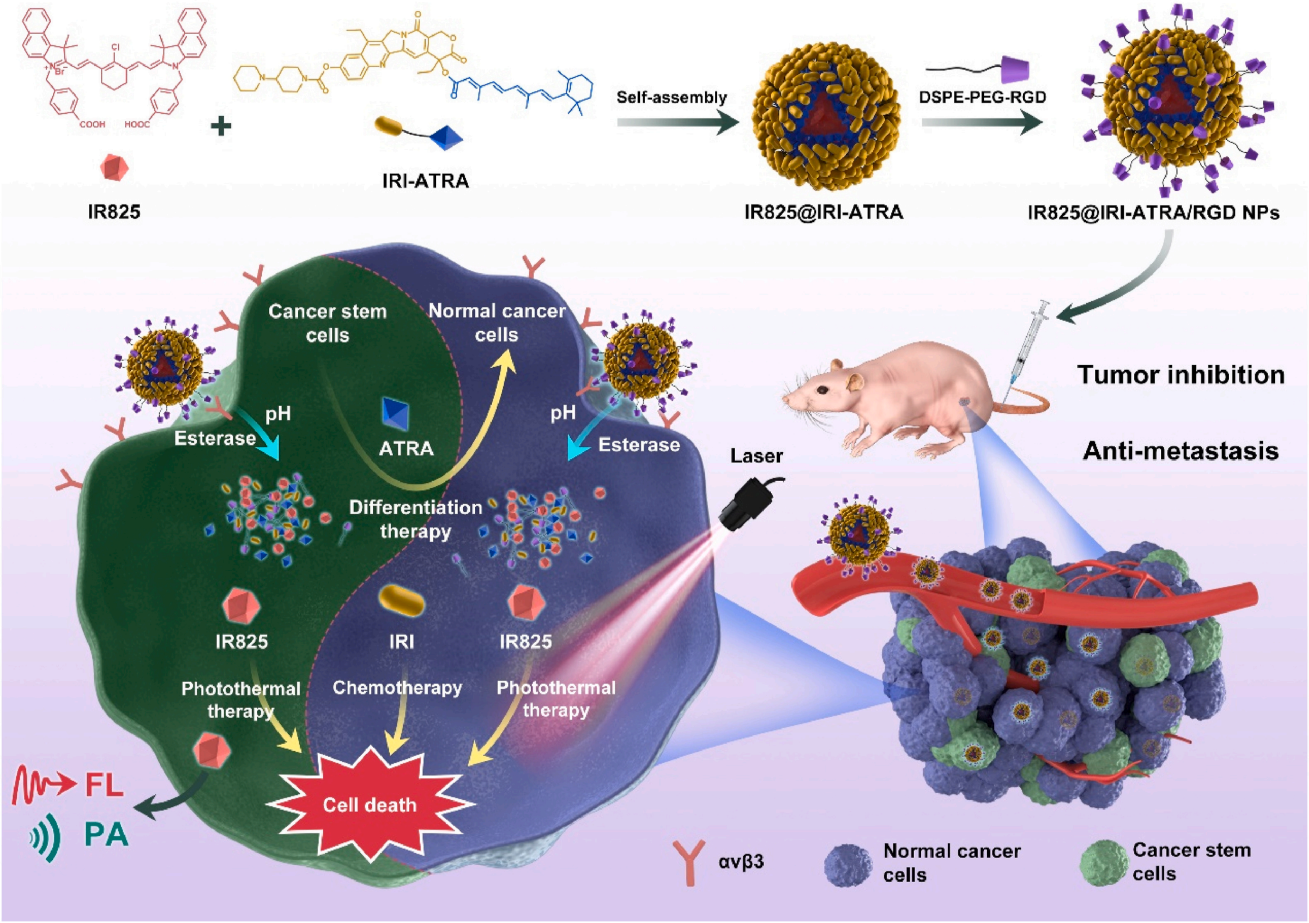
Self-assembled nanodrugs for co-delivery of ATRA, IRI, and IR825 to overcome chemoresistance by inducing differentiation of breast CSC, ATRA, and IR825 for breast CSCs. IR825@IRI-ATRA: NPs co-loaded with IR825 and IRI-ATRA; FL: fluorescence imaging; PA: photoacoustic imaging; avβ3: integrin αvβ3 *Note.* Reprinted with permission from “A small-molecule self-assembled nanodrug for combination therapy of photothermal-differentiation-chemotherapy of breast cancer stem cells,” by Xie X, Jiang K, Li B, Hou S, Tang H, Shao B, et al. Biomaterials. 2022;286:121598. (https://www.sciencedirect.com/science/article/abs/pii/S0142961222002381?via%3Dihub). © 2022 Elsevier B.V.

To achieve accelerated intracellular drug release for enhanced therapeutic efficacy, Xie et al. [96] recently developed arginine-glycine-aspartic acid (RGD)-modified nanodrugs assembled by amphiphilic drug conjugate of hydrophobic ATRA and hydrophilic IRI, which showed high stability and encapsulated a photothermal agent, heptamethine indocyanine dye (IR825, Figure 8). After cellular uptake, the encapsulated drugs were rapidly released through the ester bond degradation within the cells by responding to acid and esterase. The released ATRA elevated the sensitivity of the CSCs by inducing differentiation, leading to enhanced cytotoxicity of the released IRI toward the CSCs. In addition, IR825 not only served as a tracker for monitoring drug distribution by *in vivo* fluorescent/photoacoustic imaging but also produced a photothermal effect to boost cell death under laser irradiation. The RGD-decorated nanodrugs displayed high tumor accumulation and inhibited tumor growth and metastasis in the mouse models of triple-negative breast cancer (4T1).

To promote the differentiation of CSCs, Shamsian et al. [97] applied a combination of a differentiation-inducing agent, vorinostat (SAHA) and a Wnt/β-catenin signaling pathway antagonist, PKF118-310, which were loaded in the sphere gold NPs with human plasma protein corona. SAHA could inhibit histone deacetylase to induce differentiation of the CSCs and enforces the non-CSCs to reprogram to the CSCs, and PKF118-310 suppressed the Wnt/β-catenin signaling pathway to boost CSC differentiation. The SAHA-loaded gold NPs increased the CSC population with upregulated expression of the mesenchymal marker, Snail. In contrast, a combination of the PKF-loaded gold NPs reduced the CSC population and Snail expression.

## Nanomedicine for on-demand release of chemopotentiator and chemotherapeutic

NP-mediated co-delivery of multiple drugs has attracted mounting interest and achieved considerable success in augmenting combination therapeutic efficacy by unifying individual pharmacokinetics and maintaining drug ratio. However, a dense extracellular matrix (ECM), a non-cellular three-dimensional macromolecular network consisting of collagen, and polysaccharides such as HA, elastin, and fibronectin [98], renders a delivery hindrance for NPs to penetrate the deep region of tumor. Meanwhile, elevated interstitial fluid pressure (IFP) exhibits a big barrier to drug delivery in solid tumors [99]. Thus, dense ECM and high IFP prevent the NPs carrying drugs from reaching the CSCs that mainly reside in the hypoxic niche of tumor tissue [100, 101]. Furthermore, considering that combinatorial drugs often have distinct sites and mechanisms of action, it is a prerequisite for the nanocarriers to differentiate their unique targets and time of action to maximize the synergistic effects. In view of this, the nanocarriers engineered with on-demand drug release have been increasingly exploited based on the specific pathological signals within the tumor microenvironment and cells, such as tumor and endocytic acidities, hypoxia, high redox potential, and overexpressed enzymes [102].

**Figure 9:**
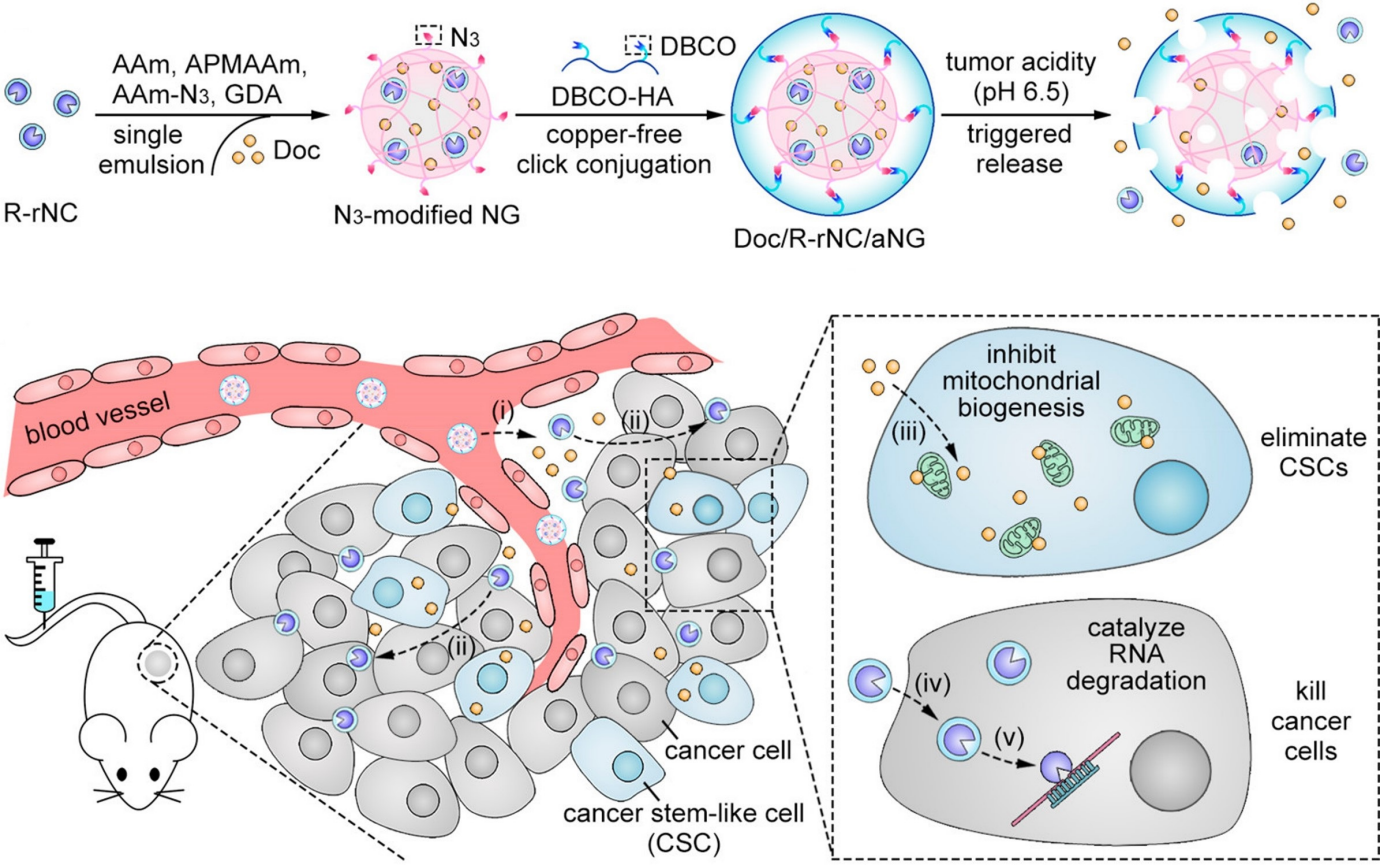
Hierarchically-assembled nanogels with tumor penetration capacity for co-delivery of RNase A and Doc to eliminate breast CSCs and cancer cells. AAm: acrylamide; APMAAm: *N*-(3-aminopropyl)methacrylamide; DBCO: dibenzocyclooctyne; Amm-N3: azide-modified AAm; GDA: glycerol dimethacrylate; NG: nanogel; aNG: acid-responsive nanogel *Note.* Reprinted with permission from “Hierarchical nanoassemblies-assisted combinational delivery of cytotoxic protein and antibiotic for cancer treatment,” by Liu M, Shen S, Wen D, Li M, Li T, Chen X, et al. Nano Lett. 2018;18:2294–303. (https://pubs.acs.org/doi/10.1021/acs.nanolett.7b04976). © 2018 American Chemical Society.

**Figure 10:**
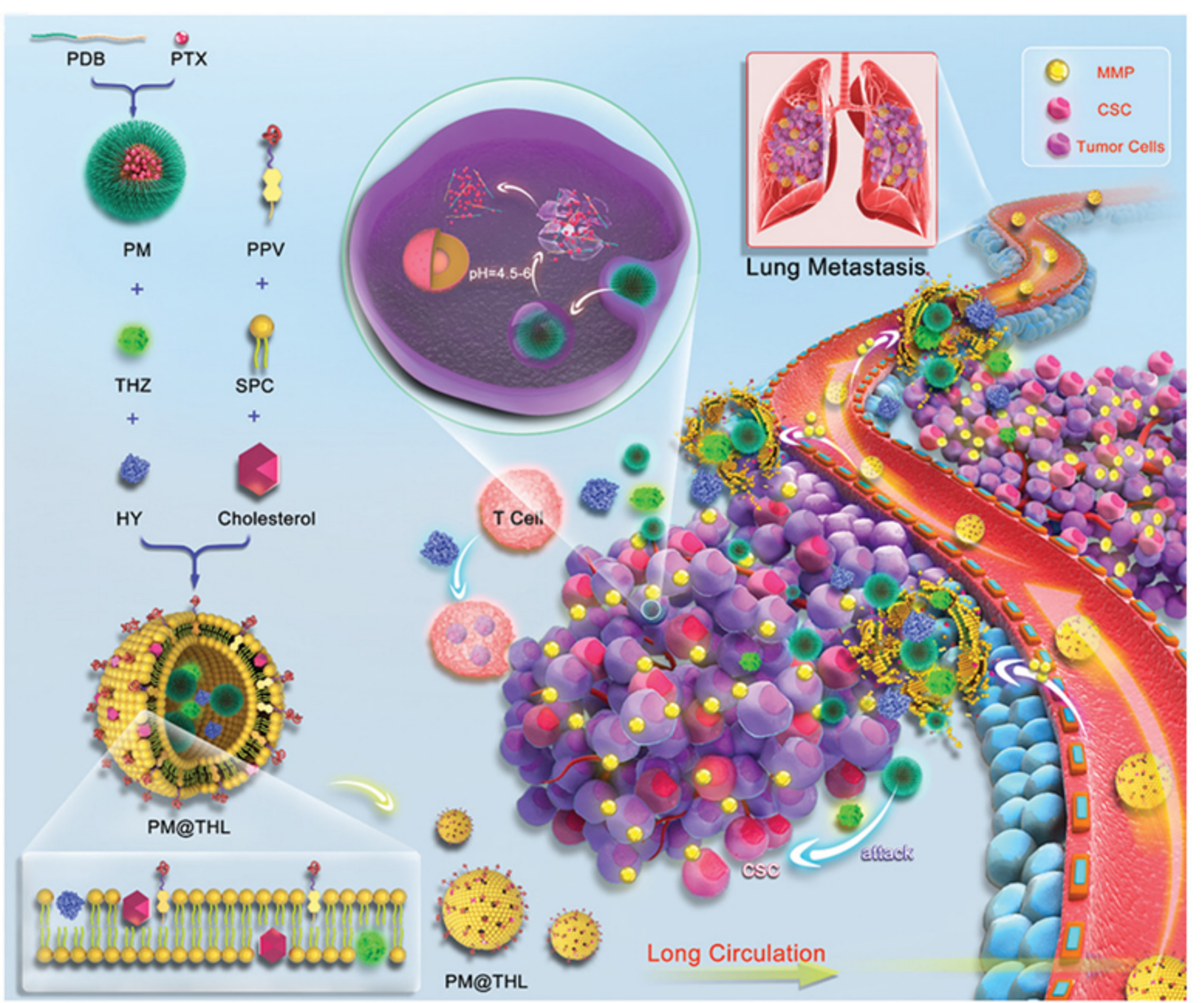
Micelle-LP double-layer structured NPs with tumor penetration capacity for co-delivery of THZ, PTX, and HY to eliminate breast CSCs and cancer cells. PDB: polyethylene glycol-block-poly[(1,4-butanediol)-diacrylate-β-N,N-diisopropylethylenediamine; PPV: methoxy polyethylene glycol-peptide (PLGLAG)-vitamin E succinate; SPC: soybean phosphatidylcholine; PM@THL: PM/THZ/HY19991-loaded hybrid liposome *Note.* Reprinted with permission from “Cocktail strategy based on spatio-temporally controlled nano device improves therapy of breast cancer,” by Lang T, Liu Y, Zheng Z, Ran W, Zhai Y, Yin Q, et al. Adv Mater. 2019;31:1806202. Erratum in: Adv Mater. 2019;31:1903844. (https://onlinelibrary.wiley.com/doi/10.1002/adma.201806202). © 2019 John Wiley & Sons, Inc.

**Figure 11:**
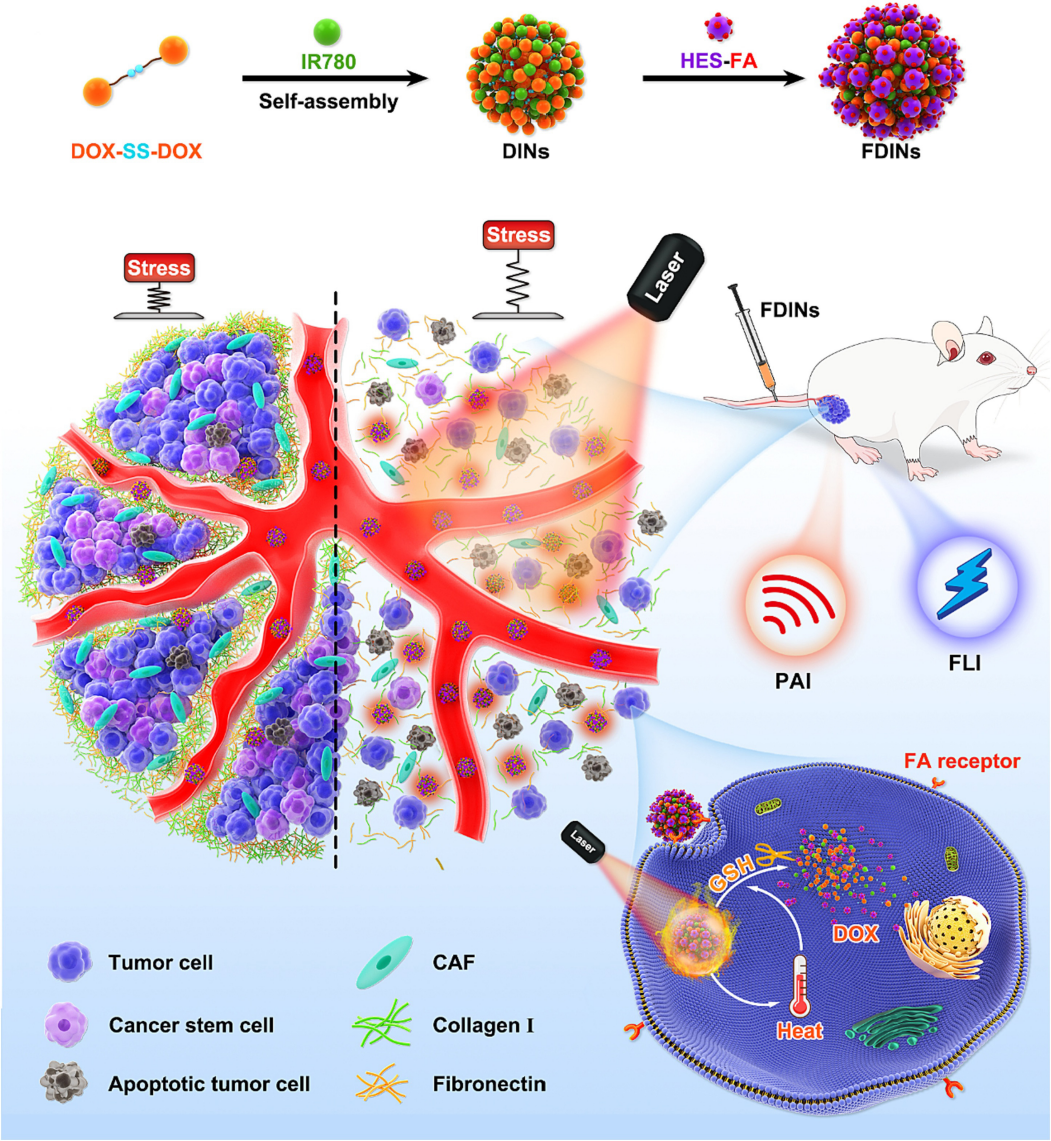
Hydroxyethyl starch NPs for co-delivery of IR780 and DOX to destruct ECM upon laser irradiation for tumor penetration to eliminate breast CSCs and cancer cells. DINs: a redox-responsive NP self-assembled by DOX prodrug of dual DOX molecules linked by a disulfide bond and IR780; FDINs: hydroxyethyl starch-folic acid stabilized DINs; PAI: photoacoustic imaging; HES: hydroxyethyl starch; FLI: fluorescence imaging; FA: folic acid; CAF: cancer-associated fibroblast *Note.* Reprinted with permission from “Hydroxyethyl starch-folic acid conjugates stabilized theranostic nanoparticles for cancer therapy,” by Wang C, Wang Q, Wang H, Li Z, Chen J, Zhang Z, et al. J Control Release. 2023;353:391–410. (https://www.sciencedirect.com/science/article/abs/pii/S0168365922008136?via%3Dihub). © 2022 Elsevier B.V.

To enhance the tumor penetration of nanocarrier for improved combination drug delivery, Liu et al. [103] developed hierarchically-assembled nanogels co-loaded with cytotoxic protein and antibiotic to overcome sequential barriers and augment antitumor efficacy by synchronously eliminating CSCs and bulk tumor cells (Figure 9). Ribonuclease A (RNase A) was embedded in a single-protein redox-responsive nanocapsule (R-rNC) with a small size of ~8 nm by interfacial polymerization, which was further encapsulated together with an anti-CSC agent, doxycycline (Doc) into a large-sized HA-decorated nanogel (~100 nm). The obtained nanogel showed preferential accumulation in the CSC-enriched MDA-MB-231 tumor tissue by the HA-mediated binding to the CD44 receptor. Under tumor microenvironmental acidity, the nanogels dissociated by the acid-activated degradation of crosslinker, and released small-sized R-rNC and small-molecule Doc, which presented increased penetration in the tumor tissue. After cellular uptake, RNase A was promptly released from R-rNC through the glutathione-triggered cleavage of crosslinker within both CSCs and cancer cells to induce cell death by catalyzing RNA degradation, while Doc eliminated the CSCs by suppressing the mitochondrial biogenesis. The hierarchically-assembled R-rNC/Doc co-loaded nanogels exhibited enhanced tumor growth inhibition with decreased CSC population on the MDA-MB-231 tumor-bearing mice. Using this large-to-small size transformation strategy, Lang et al. [104] formulated enzyme/pH dual-responsive NPs with micelle-LP double-layer architecture for co-delivery of the chemodrug PTX, the anti-CSC agent THZ and the programmed death 1 (PD1)/PD ligand 1 (PDL1) inhibitor HY19991 to treat the CSC-related resistant tumor (Figure 10). PTX was encapsulated in the acid-labile PM with a size of ~40 nm, which are subsequently loaded in the enzyme-responsive THZ/HY19991 co-loaded LPs with a size of ~100 nm. The LPs degraded in the tumor tissue containing high levels of matrix metalloproteinase-9 (MMP-9) and released PM, THZ, and HY19991 simultaneously. PM with smaller size benefited the tumor penetration and cellular uptake and released PTX in the acidic endocytic vesicles to produce a cytotoxic effect. The released HY19991 blocked the PD1/PDL1 signaling pathway to enhance T cell-mediated immune killing, while the released THZ induced increased death of the CSCs. Treatment with the LPs inhibited the growth and metastasis of MCF-7 tumor mouse models with prolonged survival by reducing the CSC proportion and promoting T-cell infiltration. Alternatively, Wang et al. [105] lately utilized photothermal therapy to destruct the ECM-abundant tumor microenvironment for enhanced tumor penetration of nanomedicine (Figure 11). A redox-responsive DOX prodrug of dual DOX molecules linked by a disulfide bond with IR780, a photothermal agent was encapsulated in hydroxyethyl starch NPs. Upon laser irritation, local temperature elevation induced the photothermal effect of IR780 bringing about the destruction of ECM as substantiated by reduced expressions of cancer-associated fibroblast-related biomarkers such as α-smooth muscle actin and fibroblast activation protein, rendering the NPs to penetrate the deep region of the tumor tissue. The DOX prodrug released the DOX molecule within both CSCs and cancer cells to induce cell death under reductive intracellular conditions. The NPs combined with laser irritation revealed preferable therapeutic efficacy in the mouse model of stroma- and CSC-enrich tumor.

**Figure 12:**
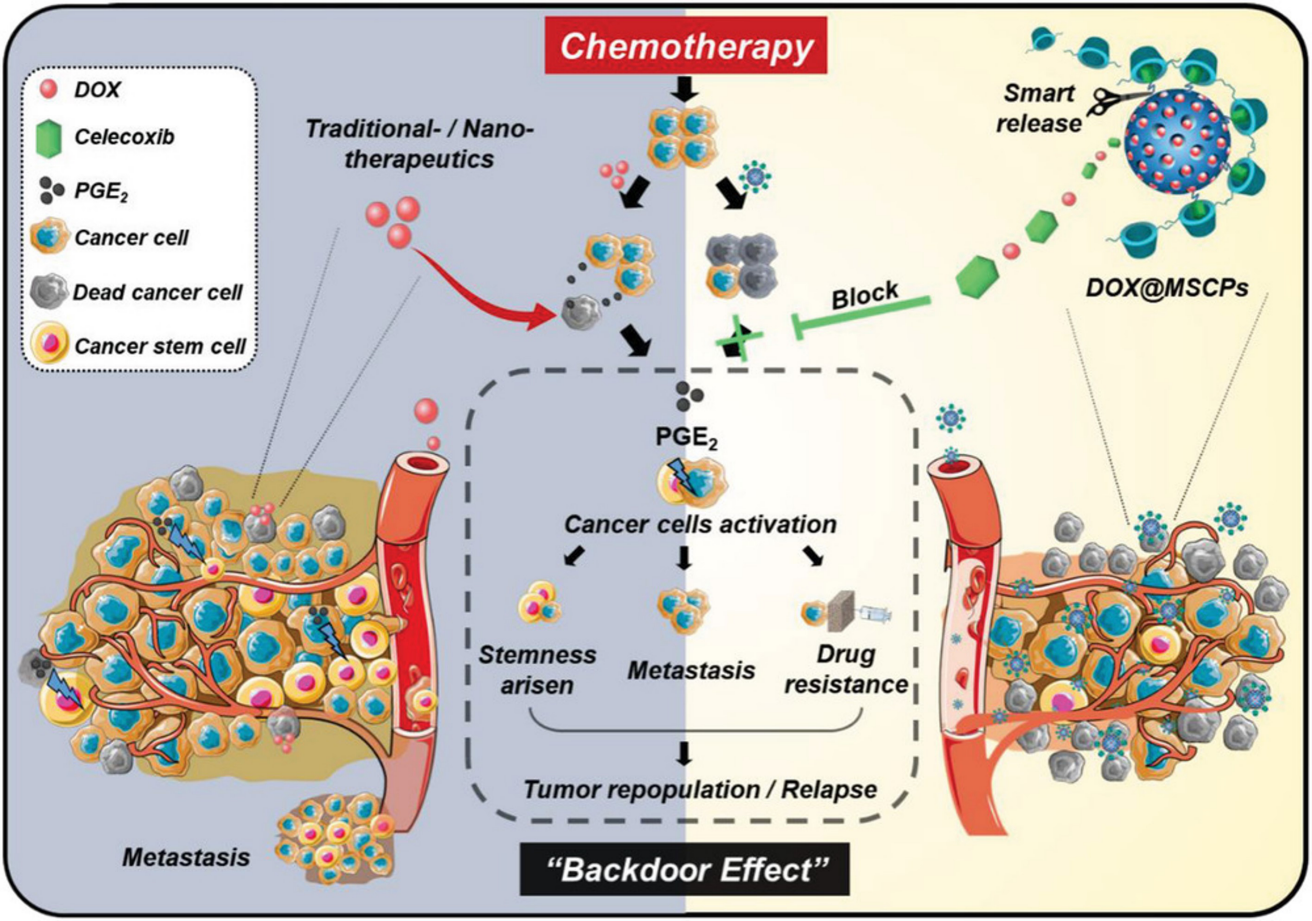
Redox-responsive MSNs for controlled release of CEL and DOX to suppress chemotherapy-induced stemness enhancement and acquired drug resistance. DOX@MSCPs: celecoxib-modified PCD-coated DOX-loaded MSNs *Note.* Reprinted from “Redox-responsive dual drug delivery nanosystem suppresses cancer repopulation by abrogating doxorubicin-promoted cancer stemness, metastasis, and drug resistance,” by Liu J, Chang B, Li Q, Xu L, Liu X, Wang G, et al. Adv Sci (Weinh). 2019;6:1801987 (https://onlinelibrary.wiley.com/doi/10.1002/advs.201801987). CC BY.

For the controlled intracellular release of combinatorial drugs by nanocarrier to overcome stemness-rooted cancer resistance, Lin et al. [106] synthesized acid/reduction dual-responsive micelles composed of polymer-drug conjugate for enhanced delivery of DTX to treat resistant prostate cancer. RUB is a small-molecule activator of miR-34 that is a key regulator of tumor suppression by repressing a variety of oncogenic signaling pathways [107]. In this study, RUB was conjugated on an acid-triggered charge-conversion polymer containing diisopropylaminoethanol (DIPAE) groups via a disulfide bond, which could self-assembled into the micelles and encapsulated DTX. After endocytosis, the micelles swelled and degraded by the protonation of DIPAE and glutathione-induced disulfide linker cleavage, leading to the rapid release of DTX and RUB. The released RUB activated miR-34a followed by downregulation of proteins associated with chemoresistance, which therefore sensitized both CSCs and tumor cells to DTX. The DTX/RUB co-loaded dual-responsive micelles showed superior anticancer efficacy in the orthotopic DTX-resistant prostate (PC3) tumor mouse model. Chemotherapeutics including DOX can upregulate cyclooxygenase-2 (COX-2), a key enzyme for the biosynthesis of prostaglandin E2 (PGE_2_), and promote the release of PGE_2_, which provokes the quiescent CSCs to proliferate and also activates dedifferentiation of bulk tumor cells to CSC-like cells leading to enhanced cancer stemness. To alleviate this chemotherapy-induced effect, Liu et al. [108] reported redox-responsive MSNs for programmable release of DOX and celecoxib (CEL), a COX-2 inhibitor (Figure 12). CEL was chemically linked on the surface of MSNs encapsulated with DOX via a disulfide bond, which was coated with poly(β-cyclodextrin) (PCD) as a gatekeeper by the interaction between CEL and CD to avoid premature release of DOX. The MSNs responsively released CEL and DOX in the cells under an intracellular glutathione-rich environment. CEL on the surface of MSNs was released relatively faster than DOX in the porous channels of MSNs. The released CEL could suppress the DOX-induced COX-2/PGE_2_ signaling pathway, resulting in the suppression of cancer stemness enhancement. The CEL/DOX co-loaded MSNs have been demonstrated capable of inhibiting DOX-activated expansion of CSCs, combating acquired drug resistance, and delaying cancer metastasis in three tumor mouse models. With the purpose of regulating drug release more precisely, Ren et al. [109] developed remote-triggered NPs with sequential drug release to reverse CSC-related drug resistance. Hollow gold NPs were employed to absorb the positively-charged DOX by electrostatic interaction and thiolated poly(amidoamine) dendrimer (PAMAM) by gold-thiolate bond linkage to condense siRNA targeting miR-21i. Upon the proton sponge effect of PAMAM, miR-21i escaped from the endocytic vesicles and released into the cytoplasm to sensitize the CSCs. Subsequent application of laser irradiation promoted the rapid release of DOX by the photothermal effect of gold NPs. The released DOX was efficient inducing apoptosis of the sensitized CSCs. Such remote-controlled sequential drug release dramatically enhanced synergistic effects both *in vitro* and *in vivo*.

**Figure 13:**
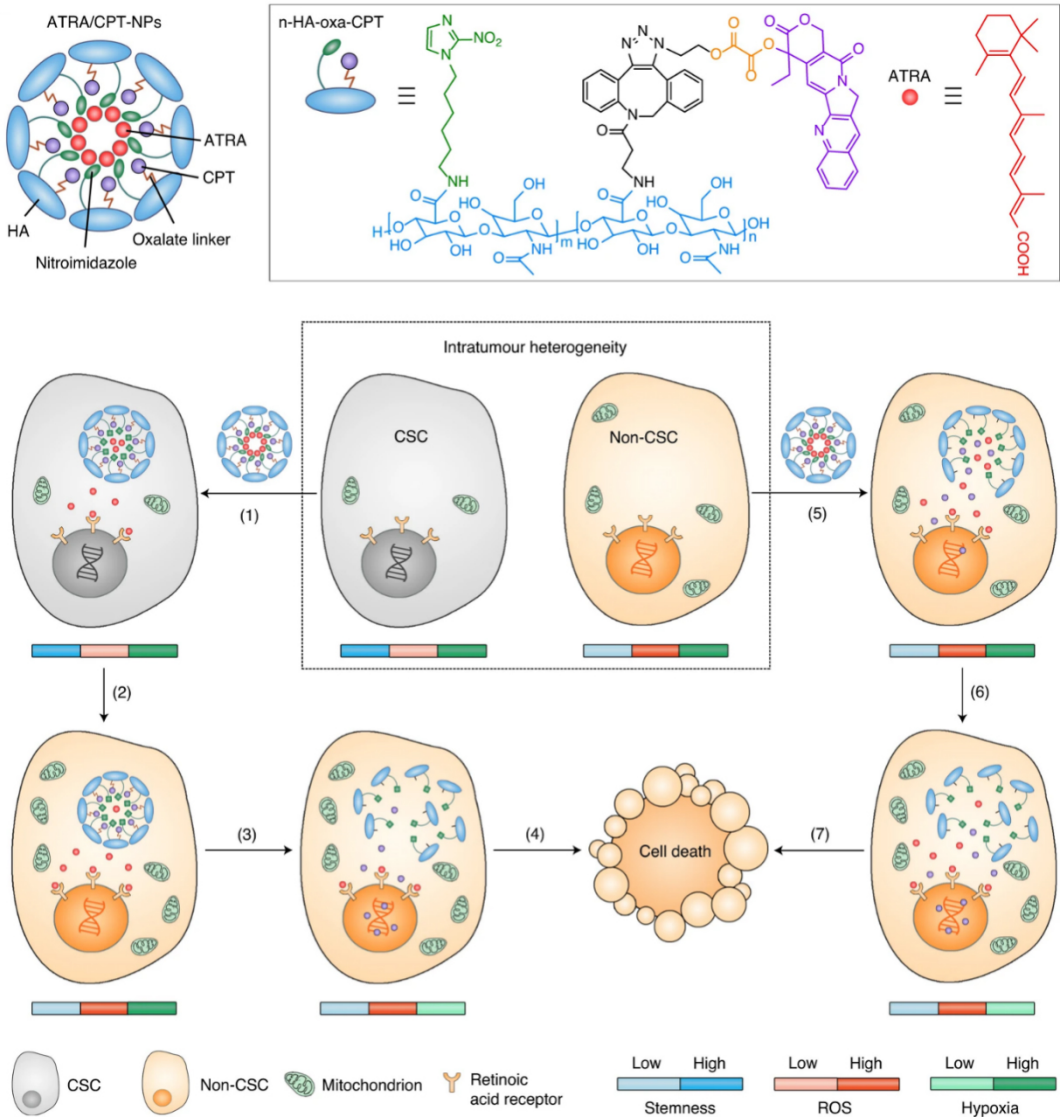
NPs with cell-specific drug release capacity to adaptively release ATRA and DOX within the CSCs and cancer cells to produce enhanced synergistic effects on overcoming stemness-associated chemoresistance. oxa: oxalate linker *Note.* Reprinted with permission from “A nanotherapeutic strategy to overcome chemotherapeutic resistance of cancer stem-like cells,” by Shen S, Xu X, Lin S, Zhang Y, Liu H, Zhang C, et al. Nat Nanotechnol. 2021;16:104–13. (https://www.nature.com/articles/s41565-020-00793-0). © 2023 Springer Nature Limited.

On-demand regulation of combinatorial drugs to take effects at the appropriate time on both CSCs and bulk tumor cells by nanocarrier upon dynamic variation of endogenous signals is of vital importance for enhanced synergistic effects on overcoming stemness-derived tumor chemoresistance. Shen et al. [110] developed cell-differentiation-regulated NPs for co-delivery and cell-specific release of ATRA and CPT to conquer the chemotherapeutic resistance of CSCs (Figure 13). CPT was chemically conjugated on the hypoxia-responsive nitroimidazole-modified HA to synthesize an amphiphilic polymer-drug conjugate via a ROS-labile oxalate linker, while ATRA was physically encapsulated in the NPs formed by the polymer-drug conjugate. The obtained ATRA/CPT co-loaded NPs supported the release of two drugs in distinct kinetics within either CSCs or bulk tumor cells. After uptake by the CSCs, ATRA was rapidly released from the NPs due to the hypoxia-activated transformation of hydrophobic nitroimidazole to hydrophilic aminoimidazole. In sharp contrast, the chemically-conjugated CPT was highly stable and minimally leaked from the NPs within the CSCs, which have a low ROS level owing to upregulated expression of free radical scavengers. This differential drug release not only allowed ATRA to take prompt effect to induce differentiation of the CSCs but also prevented both CPT efflux by CSCs with overexpression of ABC transporters and CPT-activated enhancement of stemness. Upon the CSC differentiation by the released ATRA, the mitochondrial biogenesis accelerated with the production of mitochondrial superoxide, leading to the elevation of intracellular ROS level that is positively correlated with the degree of differentiation. The increased ROS level triggered the release of CPT due to the degradation of the oxalate bond within the differentiated descendent cells with reduced resistance, which realized the released CPT to generate the effective cytotoxic effect. By comparison, CPT was rapidly released in the bulk tumor cells with high basal ROS levels to cause cell death. Such cell-specific drug release satisfied the demand for potentiating synergistic efficacy of ATRA and DOX with distinct mechanisms of action on synchronously eliminating the resistant CSCs and sensitive bulk tumor cells. Treatment with ATRA/DOX-NPs inhibited tumor growth and delayed post-surgical tumor recurrence in CSC-enriched breast tumor mouse models. Recently, Shen et al. [111] further constructed lipid-polymer hybrid NPs with the core-shell structure for cell-specific release of ATRA and DOX. The hybrid NPs were composed of the liposomal membrane loaded with ATRA and the polymeric NP encapsulated with DOX. The drug ratio could be readily modulated by changing the proportion of drug-loaded LPs and NPs in an on-demand manner. Moreover, this formulation could be generalized for the co-delivery of more drug combinations because the physical encapsulation was used for drug loading rather than chemical conjugation that depended on the unique structure of drug molecules. For precise control of drug release behavior within the CSCs and bulk tumor cells, the LP shell and NP core were engineered with hypoxia- and ROS-responsiveness, respectively, resulting in enhanced synergistic effects of ATRA and DOX. The ATRA/DOX co-loaded hybrid NPs with cell-distinct drug release kinetics suppressed the growth and metastasis of the CSC-enriched breast tumors in the mouse models.

## Conclusions

Nanomedicines of anti-CSC therapy have been summarized in terms of their potential to overcome stemness-mediated chemotherapeutic resistance. Ligand modification on the surface of nanomedicines results in the promotion of drug accumulation with elevation of drug concentration within the CSCs, which is one of the promising strategies to increase cytotoxicity of chemotherapeutic drugs against the CSCs. However, multiple mechanisms involved in drug resistance of the CSCs limit the efficacy of anticancer drugs on the CSCs despite high intracellular drug concentration achieved by nanomedicine-mediated CSC-targeted delivery. To solve this, chemosensitizers that inhibit drug efflux of the ABC transporters, downregulate the expression of anti-apoptotic proteins, and prevent EMT are combinatorially applied to overcome intrinsic resistance of the CSCs toward chemotherapy. Moreover, self-renewal signaling pathway inhibitors and differentiation-inducing drugs suppress self-renewal and proliferation of the CSCs and induce differentiation of the CSCs to their descendants with increased chemosensitivity. Nanocarrier-mediated co-delivery of chemotherapeutics with these chemopotentiators contributes to enhanced combination anticancer efficacy by improving their pharmacokinetics and maintaining drug ratio. Notably, nanocarrier-based co-delivery systems have been developed to modulate on-demand drug release by responding to specific tumor microenvironmental and intracellular signals, which supports enhanced synergistic efficacy in eliminating both resistant CSCs and bulk tumor cells. Despite these achievements of nanotherapeutic strategy to overcome stemness-associated chemoresistance, there are still several challenges that are required to fully attain clinical potential. Firstly, heterogeneity referring to the diversity of surface biomarkers on the CSCs makes calls for the needs of discovery of corresponding ligands with high affinity and the development of multiple-modified nanocarrier for enhanced targeting [112–114]. Secondly, the adsorption of serum proteins on the NPs once entering the blood forms protein corona to shield the ligands, leading to loss of targeting ability. The incorporation of PEG prevents protein corona formation to a certain extent but also activates accelerated blood clearance of nanocarrier by absorbing serum proteins [115]. Compared with surface modification, the stiffness and plasticity of NPs which are determined by the core components play a more important role in the cellular uptake of NPs [116]. Thirdly, multifarious tumor-related endogenous signals have already been explored for on-demand drug release regulated by stimuli-responsive nanocarriers in a predictable manner. However, these physiological and pathological signals, such as acidity or redox potential, change individual differences. The most efficacious or unique stimuli should be considered in the interest of achieving the ideal efficacy for clinical practice. Finally, safety evaluation of the materials and nanocarriers should be comprehensively performed such as metabolism, excretion, and long-term toxicity.

## Abbreviations

ABC: adenosine triphosphate-binding cassette
ALDH: aldehyde dehydrogenase
ATRA: all-*trans* retinoic acid
BBB: blood-brain barrier
BCL-2: B-cell lymphoma-2
CD44: cluster of differentiation 44
CDDP: *cis*-dichlorodiamminoplatinum (II)
CEL: celecoxib
COX-2: cyclooxygenase-2
CPT: camptothecin
CS: chitosan
CSCs: cancer stem-like cells
CUR: curcumin
CYP: cyclopamine
Doc: doxycycline
DOX: doxorubicin
dPG: dendritic polyglycerol
DSPE: 1, 2-distearoyl-sn-glycero-3-phosphoethanolamine
DTX: docetaxel
ECM: extracellular matrix
EMT: epithelial-to-mesenchymal transition
EpCAM^+^: epithelial cell adhesion molecule^+^
G_0_: resting phase
HA: hyaluronic acid
IONPs: iron oxide nanoparticles
IR825: heptamethine indocyanine dye
IRI: irinotecan
LP: liposome
LRP1: lipoprotein receptor-related protein 1
miRNAs: microRNAs
M-Sal: salinomycin-loaded PEG-block-poly(-caprolactone) micelle
MSN: mesoporous silica nanoparticle
MUC1: mucin 1
NPs: nanoparticles
Oct4: octamer-binding transcription factor 4
PEG: poly(ethylene glycol)
PGE_2_: prostaglandin E2
P-gp: P-glycoprotein
PLGA: poly(lactic-co-glycolic acid)
PMs: polymeric micelles
Pt: platinum
PTX: paclitaxel
RGD: arginine-glycine-aspartic acid
RNase A: ribonuclease A
ROS: reactive oxygen species
R-rNC: ribonuclease A was embedded in a single-protein redox-responsive nanocapsule
RUB: rubone
SAHA: vorinostat
Sal: salinomycin
siABCG2: small interfering RNA targeting adenosine triphosphate-binding cassette subfamily G member 2
siBCL-2: small interfering RNA targeting B-cell lymphoma-2
siBmi1: small interfering RNA targeting BMI1 proto-oncogene, polycomb ring finger
siNotch1: small interfering RNA targeting Notch receptor 1
SMA: styrene maleic acid
SN38: 7-ethyl-10-hydroxycamptothecin
Snail1: Snail family transcriptional repressor 1
Sox2: SRY-box transcription factor 2
Tar: tariquidar
THZ: thioridazine
TMZ: temozolomide
TPGS: *D*-α-tocopherol polyethylene glycol succinate
TRAIL: tumor necrosis factor-related apoptosis-inducing ligand
UA: ursolic acid
uPAR: urokinase plasminogen activator receptor
Wnt: Wnt/β-catenin signaling

## Declarations

### Copyright

© The Author(s) 2023.
